# Reconstruction of gene networks using prior knowledge

**DOI:** 10.1186/s12918-015-0233-4

**Published:** 2015-11-20

**Authors:** Mahsa Ghanbari, Julia Lasserre, Martin Vingron

**Affiliations:** Computational Molecular Biology, Max Planck Institute for Molecular Genetics, Ihnestr. 63-73, Berlin, D-14195 Germany

**Keywords:** Gene regulatory networks, Network reconstruction, Prior knowledge, Bayesian networks

## Abstract

**Background:**

Reconstructing gene regulatory networks (GRNs) from expression data is a challenging task that has become essential to the understanding of complex regulatory mechanisms in cells. The major issues are the usually very high ratio of number of genes to sample size, and the noise in the available data. Integrating biological prior knowledge to the learning process is a natural and promising way to partially compensate for the lack of reliable expression data and to increase the accuracy of network reconstruction algorithms.

**Results:**

In this manuscript, we present PriorPC, a new algorithm based on the PC algorithm. PC algorithm is one of the most popular methods for Bayesian network reconstruction. The result of PC is known to depend on the order in which conditional independence tests are processed, especially for large networks. PriorPC uses prior knowledge to exclude unlikely edges from network estimation and introduces a particular ordering for the conditional independence tests. We show on synthetic data that the structural accuracy of networks obtained with PriorPC is greatly improved compared to PC.

**Conclusion:**

PriorPC improves structural accuracy of inferred gene networks by using soft priors which assign to edges a probability of existence. It is robust to false prior which is not avoidable in the context of biological data. PriorPC is also fast and scales well for large networks which is important for its applicability to real data.

**Electronic supplementary material:**

The online version of this article (doi:10.1186/s12918-015-0233-4) contains supplementary material, which is available to authorized users.

## Background

Gene Regulatory Networks (GRNs) represent the interactions among genes which control the abundance levels of gene products that are necessary to respond to the cellular environment. GRN reconstruction from expression data is a challenging problem in systems biology, not only because it suffers from high dimensionality and low sample size, as the number of genes is generally much larger than the biological samples, but also because biological measurements are extremely noisy. A variety of computational methods have been suggested to address this problem including regression methods [1], graphical Gaussian models [2] and Bayesian Networks [3]. Despite considerable progress in the field, current methods still give relatively poor results due to the noisy and sparse nature of the data or cannot be run on large datasets. Hence, the problem is still an active field and much remains to be done to improve the reliability of the solutions without increasing the computational cost. The readers are referred to [4–7] for comprehensive reviews on the field.

Exploiting other sources of knowledge is one reasonable way to address these issues. Recent advances in biology provide various data sources such as ChIP-seq data, pathway data and sequence data, each of which can shed more light on the cellular processes underlying GRNs. For instance ChIP-seq data can reveal potential target genes for transcription factors (TFs). Each of these sources is of course limited and noisy, and only gives a partial picture of gene regulation. However, taken together, they can help build a more robust description of the regulatory mechanisms, and reduce the effects of noise and sparsity in expression data. These pieces of information can be included in the process of GRN reconstruction in the form of prior knowledge, i.e. a subjective (but non-arbitrary) belief about how the network should look like. Hence, the use of prior information in network inference is a growing trend in computational biology [8–11].

Prior knowledge can be applied by discarding edges that are a priori unwanted, and enforcing edges that are a priori wanted. However we do not always have this level of confidence, particularly in biology where associations are difficult to establish. Another way is to set a prior to 1 when an edge is wanted, and to 0 when an edge is undesirable. However not all sources of prior knowledge are reliable, and when combining several, there may be inconsistencies to resolve, so potential errors should be accounted for and uncertainty modeled. In addition, not all of the edges have the same level of confidence. For instance when using ChIP-seq data, not all potential target genes for a specific TF have the same probability to be functional. In this case, the binding affinity of TF to TF binding sites is a proper proxy for functionality which can be converted into a probability. We believe that soft priors, which represent the probability of existence of an edge, are better suited for our application.

Prior information about gene interactions in GRNs is typically converted into a prior knowledge matrix *B*, in which each entry *b*_*ij*_ represents the confidence about the existence of an interaction between two nodes *X*_*i*_ and *X*_*j*_ [9], where nodes represent genes. Entries in *B* range from 0 to 1, where 0 stands for the strongest belief in the absence of an edge and 1 for the strongest belief in the existence of an edge. If no information about the edge between *X*_*i*_ and *X*_*j*_ is available, *b*_*ij*_ is set to 0.5. How to include this prior matrix into the reconstruction process depends of course on the algorithm used to construct the GRN.

One of the most popular tools to model GRNs is Bayesian networks (BNs). A BN is a graphical representation for probabilistic relationships among a set of random variables *V*={*X*_1_,…,*X*_*n*_}. The first component of a BN is its structure *G*, represented by a directed acyclic graph (DAG). A DAG is a graph containing only directed edges and no cycles, and the skeleton of a DAG is the DAG itself where directionality has been removed. Nodes correspond to the random variables in *V* and edges encode conditional dependencies over *V*. The second component of a BN is a set of distributions {*P*_*i*_(*X*_*i*_|parents(*X*_*i*_,*G*))} that are respectively conditioned on the parents of *X*_*i*_ in *G*, where a parent of *X*_*i*_ is a node *X*_*j*_ such that the edge *X*_*j*_→*X*_*i*_ is in *G*. Together, *G* and {*P*_*i*_} define a joint probability distribution *P* over *V*, written $P(X_{1},\ldots,X_{n}) = \prod _{i}{P_{i}(X_{i}|\text {parents}(X_{i},G))}$.

A DAG *G* and a probability distribution *P* are reciprocally faithful if and only if the conditional independencies (CIs) among the variables in *V* with respect to *P* are exactly those encoded by *G*. The faithful assumption in BNs implies that there is an edge between nodes *X*_*i*_ and *X*_*j*_ in the skeleton of *G* if and only if for all *Y*⊂*V*∖{*X*_*i*_,*X*_*j*_}, *X*_*i*_ and *X*_*j*_ are conditionally dependent given *Y*. By logical negation, there is no edge between nodes *X*_*i*_ and *X*_*j*_ in *G* if and only if there exists a set of nodes *Y*⊂*V*∖{*X*_*i*_,*X*_*j*_} such that *X*_*i*_ and *X*_*j*_ are conditionally independent given *Y*. Two variables *X*_*i*_ and *X*_*j*_ are conditionally independent with respect to a probability distribution *P* given a set of variables *Y*, if *P*(*X*_*i*_,*X*_*j*_|*Y*)=*P*(*X*_*i*_|*Y*)*P*(*X*_*j*_|*Y*) which denoted as (*X*_*i*_╨*X*_*j*_|*Y*) and can be estimated from the data with a conditional independence (CI) test.

Learning methods to reconstruct the structure of BNs mostly fall into two categories: score-based methods and constraint-based methods [12]. Score-based methods search the space of all possible DAGs to identify the network which maximizes a penalized likelihood function. Such algorithms include prior knowledge naturally through a prior distribution over the structure which can also serve as penalization. However these methods are computationally expensive and do not scale well.

Constraint-based methods involve the repeated use of CI tests. Under the assumption of faithfulness, if there is no *Y*⊂*V*∖{*X*_*i*_,*X*_*j*_} such that (*X*_*i*_╨*X*_*j*_|*Y*) holds true, there is an edge between *X*_*i*_ and *X*_*j*_. The naïve algorithm decides on the presence of an edge by conditioning on all possible *Y*. However, the naïve approach scales poorly and becomes infeasible for large networks due to the super exponential growth of the number of tests with respect to the number of nodes.

Most algorithms that allow prior knowledge fall into the class of BNs. Indeed BNs can include prior information very naturally via a prior distribution over network structures. For instance Imoto et al. [8] define a prior distribution on network structures as a Gibbs distribution in which the prior knowledge is encoded via an energy function. Werhli et al. [9] have extended their work to integrate multiple sources of prior knowledge and for each source express the energy function as the absolute difference between the network structure and prior knowledge matrix. However, these algorithms are not applicable for large networks because of their complexity. Some other methods fall into the class of regularized regression where regularization is applied to regression methods to infer a limited number of edges, thereby favoring important ones [10]. One can also use prior information to define the training dataset for a supervised method which uses this information in order to guide the inference engine for the prediction of new interactions [13].

The PC algorithm [14], or PC, is a popular constraint-based method which drastically reduces the number of CI tests by avoiding unnecessary ones, thereby allowing the reconstruction of larger networks. In fact, it has been shown [15] that PC scales well for sparse graphs and that, in the case where the number of nodes is much larger than the sample size, it is asymptotically consistent for finding the skeleton of a DAG, assuming the data follows a multivariate Gaussian distribution. However, by nature, the performance of PC relies heavily on the accuracy of its inner CI tests, which is not guaranteed in the presence of limited sample size and noisy data. If erroneous decisions are made, the output of PC depends on the order in which the variables are given.

In this work, we modify the PC algorithm to include prior knowledge. We first exclude the unlikely edges and then exploit the order dependency of PC by favoring unwanted edges for early testing, thus holding wanted edges out for late testing. The resulting algorithm is referred to as PriorPC. Prior knowledge is particularly advantageous when the quality of the CI tests is questionable, for example as mentioned above when data is high-dimensional and few samples are available, as is typical for gene regulatory networks.

The area under the receiver-operating characteristic curve (ROC) noted AUROC and the area under the precision recall curve (PRC) noted AUPRC are used to evaluate all presented algorithms. Following Greenfield et al. [10], our method PriorPC is evaluated on one dataset containing *Bacillus subtilis* expression data [16], and two datasets from the DREAM (Dialogue for Reverse Engineering Assessments and Methods) challenge, and for the *E. coli* and *B. subtilis* datasets only the nodes that are linked to at least one other node in the gold standard are considered for evaluation.

Our results show that the precision of the networks obtained with PriorPC is greatly improved over that of the networks obtained with PC, for every dataset. The performance further increases as the amount of prior increases, which shows consistency of our method. Additionally, the method performs better than PC even when noisy priors are included in the prior matrix, which shows robustness. Finally, the part of the network which is not subjected to prior knowledge is not negatively affected by the prior information on other edges. We also compare our result to a recently published work [10] where they modify regression methods to incorporate the prior knowledge.

## Methods

### The PC algorithm

The original PC algorithm is an unsupervised method which consists of two main steps: building the skeleton of the graph and determining the orientation of the edges. In the remainder of this manuscript, we will consider the skeleton only, and PC will stand for the first part of the original PC algorithm.

PC takes as input a set of variables *V* and an ordering order(*V*) over *V*, and returns the skeleton of the graph *G*. It starts with a complete undirected graph, where all the nodes in *V* are connected to one another, and edges are then removed iteratively based on CIs. For every ordered pair of adjacent nodes (*X*_*i*_,*X*_*j*_), all CIs (*X*_*i*_╨*X*_*j*_|*Y*) where *Y* is a subset of all nodes adjacent to *X*_*i*_ are computed in order to find a set *Y*^∗^ such that (*X*_*i*_╨*X*_*j*_|*Y*^∗^) holds true.

*Y* is at first the empty set (zero-order test), then each variable *X*_*d*_ in turn following order(*V*) (first-order test), then all possible pairs of potentials variables (*X*_*d*_,*X*_*e*_) following order(*V*) (second-order test) and so on, until a *Y*^∗^ is identified or possible conditions have been exhausted. If a *Y*^∗^ is found, then the edge between *X*_*i*_ and *X*_*j*_ is deleted. As the algorithm proceeds, the number of adjacent nodes decreases, and fewer and fewer tests are needed. Assuming a faithful distribution to *G* and perfect CI tests, PC correctly infers the skeleton of *G* [14], regardless of order(*V*).

The worst-case complexity of PC is *O*(|*V*|^*maxo*^), where *maxo* is the maximum order reached in the algorithm. If we denote *q* the maximum number of neighbors of a node in *G*, then *maxo*∈{*q*−1,*q*} [14]. For reasons of computational effect, we set the maximum order *q* to a value of 5. For sparse networks we expect this figure to exceed the number of actually occurring higher-order interactions, and, in fact, in all cases we have studied the algorithm finished before reaching it.

Although order(*V*) determines in which order the CIs should be tested, it has no effect on the output if the CI tests are always correct. The standard choice, used in most implementations, is then the lexicographical ordering. In practice however, CI tests must be performed on the available dataset, containing a limited number of samples for all the nodes in *V*.

The distribution of the variables is assumed to be a multivariate Gaussian, so CIs can be inferred by testing for zero partial correlation [17]. Let cor(*X*_*i*_,*X*_*j*_|*Y*) be the sample partial correlation between *X*_*i*_ and *X*_*j*_ given a set *Y*⊆*V*∖{*X*_*i*_,*X*_*j*_}, obtained from any method including regression, inversion of part of the covariance matrix or recursion, and ${z(X_{i},X_{j}|Y)=} {\frac {1}{2}\log \left (\frac {1+\text {cor}(X_{i},X_{j}|Y)}{1-\text {cor}(X_{i},X_{j}|Y)}\right)}$ the Fischer’s z-transform. The null hypothesis $\mathcal {H}_{0}:\text {cor}(X_{i},X_{j}|Y) = 0$ is then rejected against the two-sided alternative $\mathcal {H}_{A}:\text {cor}(X_{i},X_{j}|Y) \neq 0$ at significance level *β* if ${|z(X_{i},X_{j}|Y)|\ \sqrt {n-|Y|-3} >} {\Phi ^{-1}\left (1-\frac {\beta }{2}\right)}$, where *Φ* denotes the cumulative distribution function of a standard normal distribution [15]. In other word, PC uses the condition $ {|z(X_{i},X_{j}|Y)|\ \sqrt {n-|Y|-3} \leq t }$ to decide whether (*X*_*i*_╨*X*_*j*_|*Y*) holds true where ${t=\Phi ^{-1}\left (1-\frac {\beta }{2}\right)}$. In this manuscript we used corpcor R package [2] to estimate the partial correlation.

The use of small-sample-sized and noisy datasets (such as biological datasets) in CI tests can induce many false positives and false negatives. Moreover, in the presence of imperfect CI tests, the output of PC also depends on the significance level *β*, which allows to tune the sparsity of the resulting network but also increases the potential for errors. Because of these inevitable mistakes, edges may be wrongly removed or kept, thereby changing the adjacency structure and affecting the edges that are considered for deletion and the CI tests that are further performed. Therefore, the output of PC does depend on order(*V*), particularly when the number of nodes is large. This dependency has a cascading effect that can lead to a drastically different skeleton, rendering PC unstable. We use this weakness to our advantage and modify the ordering to include prior knowledge or/and data-based knowledge.

### PriorPC

PriorPC injects prior knowledge into the learning process. It first defines a confidence score for each edge representing the initial belief about existence of the edge. If we know a priori that some edges do not exist in the network, removing them in the early stages of the algorithm leads to more reliable neighborhoods and to a better set of CI tests in the rest of the algorithm. Similarly if we know a priori that some edges ought to be part of the network, keeping them as long as possible can lead to different neighborhoods and therefore to a different resulting skeleton. PriorPC uses confidence score first to discard the worst edges and then to rearrange the CI tests such that edges which are less likely to reflect a real interaction are considered for CI testing first, while edges with a high belief to belong to the network are subjected to CI testing last.

#### Including prior knowledge

We introduce a confidence score for each edge indicating the initial belief of existence of the edge which can be simply the prior associated with the edge. However, we do not have prior for all edges and sometimes the prior is not correct and we need the support of data for the edge as well. We define data score *d*_*ij*_ as the normalized multiplication of two *z*-scores resulting from the deviation of the correlation *cor*(*X*_*i*_,*X*_*j*_) from the two distributions of correlations *cor*(*X*_*i*_,.) and *cor*(*X*_*j*_,.). If **C** denotes the absolute correlation matrix, the unnormalized score is written ${e_{\textit {ij}} = \left |\frac {\textbf {C}_{\textit {ij}}-\mu _{i}}{\sigma _{i}}\right | \times \left |\frac {\textbf {C}_{\textit {ij}}-\mu _{j}}{\sigma _{j}}\right |}$, where *μ*_*i*_ and *σ*_*i*_ (resp. *μ*_*j*_ and *σ*_*j*_) are the mean and standard deviation of the correlation values between *X*_*i*_ (resp. *X*_*j*_) and all other variables. This is similar to the CLR score ${\sqrt {\left (\frac {\textbf {M}_{\textit {ij}}-\mu _{i}}{\sigma _{i}}\right)^{2} + \left (\frac {\textbf {M}_{\textit {ij}}-\mu _{j}}{\sigma _{j}}\right)^{2}}}$ [18] with correlation instead of mutual information. The data score is then obtained using ${d_{\textit {ij}} = \frac {e_{\textit {ij}}}{\sqrt {e_{\textit {ii}}}\sqrt {e_{\textit {jj}}}}=\left |\frac {\textbf {C}_{\textit {ij}}-\mu _{i}}{\textbf {C}_{\textit {ii}}-\mu _{i}}\right | \times \left |\frac {\textbf {C}_{\textit {ij}}-\mu _{j}}{\textbf {C}_{\textit {jj}}-\mu _{j}}\right |}$. For the data score to be high, the observed correlation between *X*_*i*_ and *X*_*j*_ must be far from the average correlation involving *X*_*i*_ and from the average correlation involving *X*_*j*_.

We define the confidence score *s*_*ij*_ of an edge *X*_*i*_−*X*_*j*_ as *s*_*ij*_=*α*×*b*_*ij*_+(1−*α*)×*d*_*ij*_, where 0≤*alpha*≤1, *b*_*ij*_ is the prior associated with the edge and is directly read from the prior matrix *B*, and *d*_*ij*_ is a data-based score. While *b*_*ij*_ encodes our belief in the existence of the edge, *d*_*ij*_ indicates how well the edge is supported by the data. To have a high confidence score, an edge must be supported by the prior or the data. Which source matters most depends on *α*.

#### Discarding the worst edges

Edges are ranked by decreasing confidence score *s*_*ij*_. All edges after the top *N*_*E*_≃3×|*E*|, where |*E*| is the number of expected edges, are discarded. This number stems from the idea that the network should be sparse [19], and from the three tier structure of the algorithm developed in the next section. This bold step replaces the zero-order CI tests in PC. Indeed, the zero-order CI tests can also be seen as a deletion step where edges are ordered by decreasing marginal correlation rather than confidence score, and deleted one by one until the CI test reaches the desired threshold. This step is also comparable to a high penalty on the number of edges.

#### 3-tier structure

After discarding the worst edges, the remaining *N*_*E*_ edges are divided into three categories. We convert PC into a 3-tier algorithm, where in each tier a specific category of edges is tested for CIs. We consider the top $\frac {1}{3}$ of *N*_*E*_ edges to be strong candidates, the bottom $\frac {1}{3}$ to be weak candidates, and the remaining $\frac {1}{3}$ to be average candidates. While PC runs all zero-order CI tests for all edges, then proceeds with the first-order CI tests and so on, PriorPC performs all CI tests of order 1 to 5 for all weak candidates first, then for average candidates, and finally for strong candidates.

If the confidence score of a candidate edge and the subsequent group in which it falls is a good indicator, 3-tier PC can remove more false edges, and faster. For instance, if there is a false edge *X*_*i*_−*X*_*j*_ for which (*X*_*i*_╨*X*_*j*_|*Y*=*Y*_1_,*Y*_2_) holds true, PC must perform several unnecessary first-order and second-order CI tests before getting to the relevant one. This is not only computationally expensive but also undesirable, because these unnecessary CI tests can cause multiple errors and lead to strong effects as discussed previously. Instead PriorPC removes the worst candidates at the very beginning, and the weak candidates earlier than the other candidates. This also leads to a more reliable neighborhood and CI tests when assessing strong candidates.

### Edge ranking by bootstrapping

To build smooth ROC and PR curves, the algorithm can provide as output a ranking of the edges. Note that this ranking is not the same as the one given by the confidence score. Instead this ranking can be seen as a ranking a posteriori, where prior information and data structure have both been processed by the algorithm.

PC and PriorPC do not naturally allow for such a ranking. To remedy that issue, we have chosen to apply bootstrapping and to post-rank the edges by their frequency of appearance when running a chosen algorithm several times. *K* sub-datasets *D*_*k*_ are constructed from the original dataset *D* using bootstrapping (i.e. sampling with replacement) and then a chosen algorithm is applied to all *K* datasets. If *K*-fold bootstrapping is applied to PC for example, *K* networks are obtained, and an edge can appear any number of times between 0 and *K*. This number is used to create a ranking a posteriori of the edges and to produce the desired ROC and PR curves.

We set *K* to 20 for all experiments. One could use the confidence score of edges to break the ties, however we rank them lexicographically. Note that, to produce a network in the first place, a threshold for the CI tests is required. As detailed in Supplementary Material Section 2 (see Additional file 1), this threshold was fixed to 0.1 for all experiments and optimized neither for PriorPC nor for each data set.

### Synthetic prior knowledge

For each experiment and for each dataset, the prior information matrix *B* is simulated from the gold standard network available depending on the needs. To assign a true prior to an edge *X*_*i*_−*X*_*j*_, we check the existence of that edge in the gold standard network. If the edge is present, the prior *b*_*ij*_ is randomly sampled from (0.5,1], otherwise *b*_*ij*_ is randomly sampled from [0,0.5). To assign a non-informative prior to *X*_*i*_−*X*_*j*_, *b*_*ij*_ is set to 0.5.

## Results and discussion

### Datasets

For the evaluation of the PriorPC, we used three different datasets. Two of them are from DREAM challenge. The DREAM challenge is an annual reverse engineering competition with the aim of fair comparison of network inference methods. Participants are asked to generate a network structure for each dataset with a confidence score for each edge. In the following, we explain the three datasets in more detail. Note, each dataset contains both time-series data and steady state data and we only use the steady state data. We used the gold standard of each data set to synthesize prior knowledge.

- A synthetic dataset from the DREAM4 competition [20–22]. The data consists of 100 genes where any gene can be a regulator. The gold standard contains 176 interactions. The normalization was done by the DREAM organizers.
- A real dataset from the DREAM5 competition [21]. The data includes a compendium of microarray experiments measuring the expression levels of 4511 *E. coli* genes (344 of which are known transcription factors) under 805 different experimental conditions. Normalization was done using RMA [23]. DREAM5 challenge also provides a gold standard mainly come from RegulonDB [24] consisting of 2066 established gene regulatory interactions.
- A set of 269 expression measurements of *B.subtilis* genes in response to a variety of conditions [16]. Greenfield et al. [10] normalized the data and compilated the overlapping probes into intensities and we used the data provided by them. The gold standard comes from SubtiWiki [25, 26] which is repository of information for *B.subtilis* contains 2422 interactions.

Note that PC is not feasible for large networks with a small threshold for the CI tests and we compare PriorPC to PC-lite. PC-lite is a variation of PC that removes edges with low correlation and keeps the *N*_*E*_ edges with the highest correlation instead of doing zero-order tests (the step of discarding the worst edges of PriorPC), and then applies PC to these edges only. As it is shown in Supplementary Material Sections 1 and 2 (see Additional file 1), PC-lite always outperforms PC. We set *N*_*E*_ to 600, 7000, 7000 for DREAM4, *E. coli* and *B.subtilis* respectively. In addition, in the Supplementary Material Section 1 (see Additional file 1) we compare the results of the various steps taken between PC and PriorPC in order to see the effect of each step.

### Effect of the parameter *α*

**Fig. 1:**
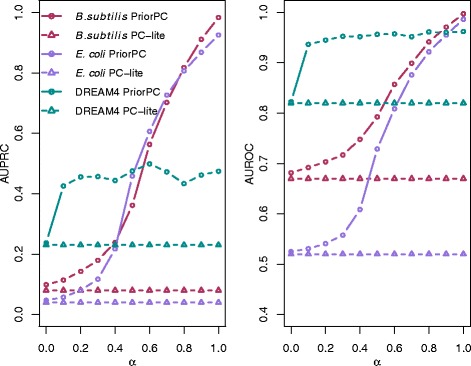
Performance of PriorPC against *α*. The left subplot shows AUPRC, while the right subplot shows the AUROC. PC-lite is plotted with triangles, while PriorPC is plotted with circles. The different colors represent the different datasets. For PriorPC, all edges have a true prior. PriorPC outperforms PC-lite and its performance increases with *α*

The value of *α* determines the degree of influence of the prior knowledge in the ranking of the edges. While *α*=1 means ranking the edges using prior knowledge only, *α* = 0 means using data only. Figure 1 shows the performance of PriorPC for different values of *α*. In this experiment, the prior matrix *B* contains only true priors, i.e. priors sampled in (0.5,1] for present interactions and in [0,0.5) for absent interactions.

PriorPC performs well above PC-lite, even though priors were simply sampled between [0,0.5) or (0.5,1]. Increasing the value of *α* leads to a better performance. This indicates that not all of the edges are well supported by the data and therefore increasing the effect of the prior improves the algorithm. This also emphasizes the value of integrating prior knowledge where data is sparse and noisy.

Note that PriorPC with *α*=1 does not perform perfectly. Indeed, the prior is used to reorder the CI tests, but it has no effect on the CI tests themselves. Therefore, it is not possible to reconstruct the real network unless data supports it. We provide the list of the edges of DREAM4, *E. coli* and *B.subtilis* for *alpha*=0.5 (see Additional files 2, 3 and 4). These lists can be used as input for Cytoscape [27] to visualize the corresponding networks.

### Effect of the amount of prior knowledge

**Fig. 2:**
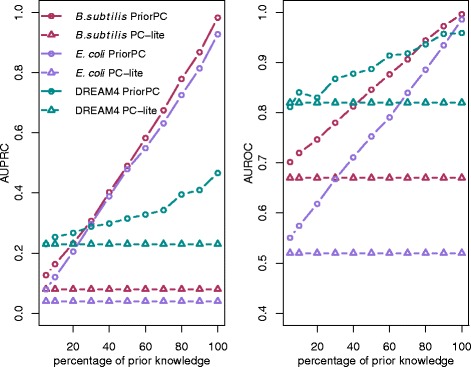
Performance of PriorPC against the percentage of edges with a prior. The left subplot shows the AUPRC, while the right subplot shows the AUROC. PC-lite is plotted with triangles, while PriorPC is plotted with circles. The different colors represent the different datasets. PriorPC outperforms PC-lite and its performance increases with the percentage of edges with a true prior

In order to assess the effect of the prior on the resulting network, the algorithm was given different amounts of prior knowledge. Initially, 5 % of the edges were randomly selected and assigned a true prior as stated in Section “Synthetic prior knowledge”. For all other edges, the prior was set to 0.5. The percentage of the edges with a true prior was then gradually increased until it reached 100 %. Figure 2 shows the results for *α*=1.

Here again, PriorPC performs well above PC-lite, even though priors were simply sampled between [0,0.5) or (0.5,1]. For each dataset, the more prior is included, the better the network can be recovered. This indicates that PriorPC is consistent.

### Effect of the prior knowledge on the edges without prior

**Fig. 3:**
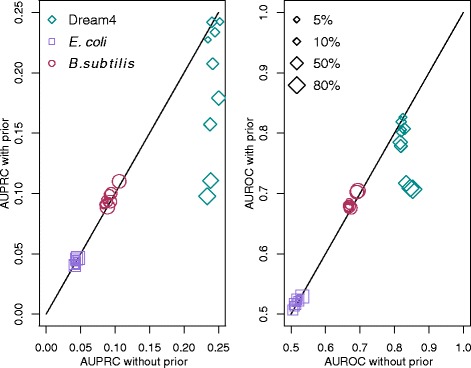
Comparison between PC-lite and PriorPC on neutral edges. Neutral edges are edges which are not subjected to prior knowledge. The left subplot shows the AUPRC, while the right subplot shows the AUROC. The x-axis shows the performance of PC-lite, the y-axis the performance of PriorPC. Each datapoint corresponds to a different amount of edges with a true prior from 5 % to 80 %. For PriorPC, *α*=1. Results are comparable, overall neutral edges are not negatively affected by the prior

The prior, even if it is incomplete and only concerns a few edges, may influence the complete network. We refer to the edges that do not have a prior as neutral edges. To assess the influence of the prior on neutral edges, 5 % of the edges were randomly sampled and assigned a true prior. This experiment was repeated for increasing percentages, until 80 % edges were selected. The results were then compared with PC-lite but this time separately for the neutral edges and for the edges with prior. Figure 3 shows the results for *α*=1.

The results show that for real data, parts of the network which are not subjected to the prior do not suffer from the prior. For DREAM4 data, using a high amount of prior leads to a performance decrease on the neutral edges, it is unclear why. The rest of the time, the performance is just as good as that of PC-lite.

### Robustness to erroneous priors

Biological prior knowledge can come from different sources including ChIP-seq data, protein-protein interaction data and literature, which can all contain false information. Methods for integrating prior knowledge should therefore be robust to errors.

**Fig. 4:**
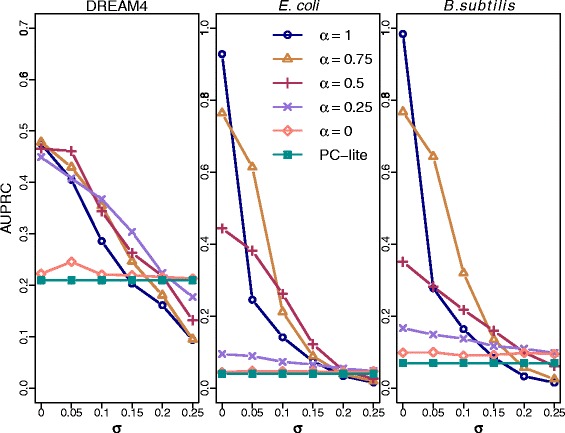
Performance of PriorPC against *σ* for various *α*s in terms of AUPRC. All edges have a true prior. Gaussian noise is added to all priors with various standard deviations *σ*. The different colors represent the result for various *α*s. The performance of PC is plotted in green and with full squares for comparison. For small standard deviations, PriorPC performs better than PC-lite. This effect is not seen for large standard deviations since most priors are flipped

**Fig. 5:**
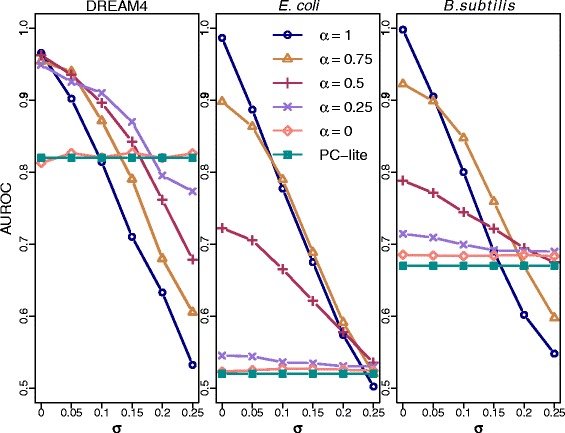
Performance of PriorPC against *σ* for various *α*s in terms of AUROC. All edges have a true prior. Gaussian noise is added to all priors with various standard deviations *σ*. The different colors represent the result for various *α*s. The performance of PC is plotted in green and with full squares for comparison. For small standard deviations, PriorPC performs better than PC-lite. This effect is not seen for large standard deviations since most priors are flipped

In order to assess the robustness of the algorithms to erroneous prior information, a noisy prior $\hat {b_{\textit {ij}}}$ was assigned to all edges. Let *e*_*ij*_∼*N*(0,*σ*) and *b*_*ij*_ be the true prior for the edge *X*_*i*_−*X*_*j*_, then $\hat {b_{\textit {ij}}}=b_{\textit {ij}}-|e_{\textit {ij}}|$ if *X*_*i*_−*X*_*j*_ is a true edge and $\hat {b_{\textit {ij}}}=b_{\textit {ij}}+|e_{\textit {ij}}|$ if *X*_*i*_−*X*_*j*_ is not a true edge. We assigned noisy prior to all edges with various standard deviations *σ*. Clearly, the effect of the amount of noise (*σ*) depends on the value of *α*. Figures 4 and 5 show the effect of noise on the AUPRC and the AUROC, respectively, for different values of *α*. The results indicate that PriorPC is robust to a reasonable amounts of noise. Clearly, the higher the amount of noise, the worse the performance. Naturally, the results are less sensitive to noise for smaller values of *α*. Indeed, when *α* is small, PriorPC is still better than PC. Based on the Figs. 4 and 5, if the reliability of the prior is not well known, we recommend *α*=0.5 because this choice leads to a gain in prediction quality up to *σ*=0.15.

For a fair comparison, we also followed the experimental set-up given in [10]. 50 % of the true edges were randomly selected and were given a prior in (0.5,1]. Then, different numbers of the remaining edges were randomly selected and given a true prior which was then flipped to introduce errors using *b*_*ij*_=1−*b*_*ij*_. The resulting AUROC and AUPRC can be seen in Supplementary Material Section 3 (see Additional file 1) which shows that PriorPC is robust to false prior up to a ratio of true priors to false priors between 1:5 and 1:10, depending on the value of *α*.

### Comparison of PriorPC to MEN and BBSR

Recently published work [10] suggests two methods to use prior knowledge. For both methods, they limited the number of potential regulators for each gene to the union of the 10 highest-scoring predictors based on tlCLR and all predictors with prior knowledge. The first method called MEN (Modified Elastic NET) is a modification of Elastic Net where prior knowledge is expressed as a modifier of the *l*1 constraint incurred on each single regression coefficient. This leads to less shrinkage on the regression coefficient corresponding to a putative regulation.

The second method called BBSR (Bayesian best subset regression) is based on Bayesian regression with a modification of Zellner’s *g* prior. In this framework the prior on the regression coefficients follows a multivariate Gaussian distribution centered at an initial guess with the empirical covariance matrix that is scaled by a chosen factor *g*, where *g* encodes the belief about the initial guess. They extend the original formulation of *g* and define a vector with one entry per predictor to allow for different levels of confidence for different entries in the initial guess. They use a criterion based on Bayesian Information Criterion (BIC) to select the final model. Since it is not feasible to compute all regression models, they reduce the set of potential regulators to the 10 best predictors based on average expected BIC. For both methods, bootstrapping is applied in order to provide a final ranking of the edges.

BBSR and MEN take as input both steady state data and time series data and the output is a matrix with confidence level for directed edges. For a fair comparison we just take the skeleton and assign the highest confidence of corresponding directed edges to undirected edge.

The prior used in BBSR and MEN is not probabilistic, instead it is a hard score stating the strength of belief in the presence of an edge, with 1 for belief and 0 for no belief (no belief in the sense of no opinion, which is similar to a probability of 0.5). The score 1 is assigned to the edges found in the gold standard network only. The rest of the edges are assigned the score 0. The two methods are compared with their respective core methods and with state-of-the-art algorithms which do not contain any prior information. In each case, the inclusion of prior knowledge improves the accuracy of the inferred network.

We compare PriorPC to these two methods. Table 1 and 2 show the AUPRC and AUROC results, respectively, from MEN and BBSR for different (default) parameters corresponding to the low and high use of prior as well as the results of PriorPC for two different values of *α*. For the sake of comparison, we followed Greenfield et al. [10]: 50 *%* of the true interactions in the gold standard network are selected and assigned a true prior (1 for MEN and BBSR, a random probability in (0.5,1] for PriorPC).

**Table 1:** Comparison of MEN, BBSR and PriorPC in terms of AUPRC

	DREAM4	*E. coli*	*B.subtilis*	Using TS
MEN_low	0.48	0.201	0.218	Yes
MEN_high	0.571	0.347	0.369	Yes
BBSR_low	0.44	0.196	0.269	Yes
BBSR_high	0.519	0.359	0.394	Yes
PriorPC (*α*=1)	0.328	0.413	0.392	No
PriorPC (*α*=0.75)	0.341	0.336	0.303	No

For all three methods, 50 *%* of the edges present in the gold standard network were randomly selected and assigned a true prior (1 for MEN and BBSR, a random probability in (0.5,1] for PriorPC). For PriorPC, *α* is given in brackets. MEN and BBSR also use time-series(TS) data. Results are comparable across the three algorithms

**Table 2:** Comparison of MEN, BBSR and PriorPC in terms of AUROC

	DREAM4	*E. coli*	*B.subtilis*	Using TS
MEN_low	0.908	0.768	0.828	Yes
MEN_high	0.912	0.776	0.842	Yes
BBSR_low	0.872	0.675	0.791	Yes
BBSR_high	0.86	0.719	0.793	Yes
PriorPC (*α*=1)	0.887	0.753	0.835	No
PriorPC (*α*=0.75)	0.885	0.71	0.801	No

For all three methods, 50 *%* of the edges present in the gold standard network were randomly selected and assigned a true prior (1 for MEN and BBSR, a random probability in (0.5,1] for PriorPC). For PriorPC, *α* is given in brackets. MEN and BBSR also use time-series(TS) data. Results are comparable across the three algorithms

The results show that on average PriorPC performs as well as BBSR and MEN even without the use of time-series (TS) data and merely using soft prior. Note that none of PriorPC’s parameters were tuned. PriorPC is also fast and one bootstrap takes 1:08, 39:34, 6:01 min for DREAM4, *E. coli* and *B. subtilis* respectively, when *α*=1 (3.1GHz Intel Core).

## Conclusion

We presented PriorPC, a variation of the PC algorithm which uses prior knowledge. PriorPC defines a confidence score for each edge reflecting the prior knowledge. Based on this confidence score, PriorPC discards the most unlikely edges. This leads to a more reliable neighbourhood for doing the CI tests later in the algorithm. In the next step it exploits the order dependency of PC by rearranging the CI tests in order to favor less probable edges for early testing and to keep more likely edges for late testing. This dependency of PC is due to sparse and noisy data which affects negatively the performance of the CI tests. The larger the number of variables, the more impact the order has.

PriorPC uses soft priors which assign to edges a probability of existence, rather than hard priors which give edges an existence state. We believe soft priors are more desirable as they can summarize the level of uncertainty the source associates with the edge, and the level of uncertainty associated with source itself.

PriorPC is evaluated on three different datasets. Although parameters are never tuned at any point of the experiments, PriorPC produces a significant improvement in structural accuracy over PC for every dataset at hand. This improvement consistently increases with the amount of prior. Moreover, in the presence of partial prior knowledge, the part of the network that has no prior is not badly affected by the partial prior.

The robustness of the algorithm to noise in the prior matrix, which is not avoidable in the context of biological data, was tested. The results show that in the presence of noisy priors, PriorPC still performs better than PC up to a level of noise of 0.15. This transition level depends on how strong the dependency to prior knowledge is, i.e. how high *α* is. Similarly, if priors are flipped (i.e. false) rather than noisy, PriorPC performs better up to a ratio of true priors to false priors between 1:5 and 1:10. Again this ratio depends on *α*. In practice, if the reliability of the available prior knowledge is questionable, it is advisable to use *α*=0.5. This choice leads to prediction gain for reasonable amount of noise and false prior.

PriorPC is fast and scales well while most Bayesian network reconstruction methods which use prior knowledge are not feasible for large networks. These methods are mostly in the class of score-based methods and usually involve Markov-Chain-Monte-Carlo algorithm which is computationally expensive.

Synthetic priors were generated in order to assess the algorithm. Positive priors were randomly sampled between (0.5,1] and negative priors between [ 0,0.5). As future work, it would be interesting to see how performance changes when using real priors. Prior knowledge can be obtained from different sources including experimental data like ChIP-seq data, pathway databases such as KEGG, protein-protein interaction data and even information derived from relevant literature. All theses sources of information can be included in a prior knowledge matrix representing the aggregated belief about gene interactions.

Employing new experimental data for validation and testing of the algorithm is difficult because it would require yet another level of experimental data as a gold standard. This is why in this paper we have focused on synthetic data and on the dependence of the results under different kinds of perturbations. It remains an open question how to translate this to the real world, in that we cannot tell what noise level, e.g., a real ChIP-seq experiment would correspond to.

In this manuscript, we focused on the structure of the network and did not consider edge directionality. The accuracy of direction assignment critically depends on the structure. After a better skeleton is obtained, the second phase of the original PC algorithm can be applied to partially assign directions. It is also possible to adopt a hybrid (constraint/score) algorithm [28] by first using PriorPC to obtain an estimate of the GRN structure and then use a score-based method to assign the direction and find the final network.

## Additional files

Additional file 1 **Supplementary material.** (PDF 180 KB)

Additional file 2 **List of the edges for DREAM4 data with** ***a*** ***l*** ***p*** ***h*** ***a*** **=0** ***.*** **5**. (TEXT 3 KB)

Additional file 3 **List of the edges for** ***E. coli*** **data with** ***a*** ***l*** ***p*** ***h*** ***a*** **=0** ***.*** **5**. (TEXT 82 KB)

Additional file 4 **List of the edges for** ***B.subtilis*** **data with** ***a*** ***l*** ***p*** ***h*** ***a*** **=0** ***.*** **5**. (TEXT 115 KB)

## Abbreviations

AUROC: Area under the receiver-operating characteristic curve
AUPRC: Area under the precision recall curve
BN: Bayesian network
CI: Conditional independence
DAG: Directed acyclic graph
DREAM: Dialogue for Reverse Engineering Assessments and Methods
GRN: Gene regulatory network
PRC: Precision recall curve
ROC: Receiver-operating characteristic curve
TF: Transcription factor
