# Supplementary material for: Reconstruction of gene networks using prior knowledge
Source: BMC Syst Biol. 2015 Nov 20;9:84. doi: 10.1186/s12918-015-0233-4 (PMC4654848; doi:10.1186/s12918-015-0233-4)
Supplement: Supplementary file 1 — Supplementary material. (PDF 180 KB) [file 12918_2015_233_MOESM1_ESM.pdf]

Supplementary Material: Reconstruction of gene networks using prior knowledge

Mahsa Ghanbari , Julia Lasserre , Martin Vingron
Max Planck Institute for Molecular Genetics,
Ihnestrasse 63-73 D-14195 Berlin, Germany

1 From PC to PriorPC

In this section, the results of the various steps taken between PC and PriorPC in order to see the effect of each step. Note that, in this section, bootstrapping was not used because it would take too long for PC.

- The first step is to rank the edges based on their correlation and to use this ordering instead of the lexicographic ordering in PC. We refer to this algorithm as OPC (ordered PC).
- The second step is to remove edges with low correlation and keep the N_E edges with the highest correlation instead of doing zeroth-order tests, and apply PC to these edges only. We refer to this algorithm as PC-lite.
- We refer to the combination of OPC and PC-lite as OPC-lite.
- Finally, PC-prior is the algorithm that discards the worst edges based on the prior and then applies PC on the top N_E edges.

In this experiment, the prior matrix B contains only true priors, i.e. priors sampled in $(0.5, 1]$ for present interactions and in $[0, 0.5)$ for absent interactions.

PC, OPC, PC-lite, OPC-lite, PC-prior and PriorPC with different values of α were applied to the three datasets, all using the same threshold for the CI tests. Table 1 compares the number of true positives (TP), the number of false positives (FP) and the F1 scores (the harmonic mean of recall and precision) obtained for each algorithm for a CI threshold of 0.1. The results show that the most effective steps are to discard the worst edges at the very beginning and to include prior knowledge.

The difference between PC and OPC lies strictly in how edges are ordered: by lexicographical order or correlation. Results are comparable on DREAM4 data, but OPC clearly wins for two datasets (*E. coli* and *B.subtilis*). Similarly, the difference between PC-lite, OPC-lite and PriorPC with $\alpha = 0$ lies (mostly but not strictly as PriorPC also has a tier-structure) in how edges are ordered: by lexicographical order, marginal correlation or data score d_{ij}. Results are comparable, with an advantage for PriorPC with $\alpha = 0$ on *B.subtilis* data.

The difference between PC and PC-lite, or between OPC and OPC-lite, lies strictly in the removal of the worst edges at the very beginning. The results are much better for PC-lite compared to PC, and for OPC-lite compared to OPC, in all three datasets.

The difference between PriorPC with $\alpha = 0$ and PriorPC with $\alpha > 0$ lies strictly in the use of prior knowledge. Although PriorPC with $\alpha = 0$ was already consistently better than PC, OPC, PC-lite and OPC-lite, the results are further greatly improved with $\alpha > 0$, on all three datasets.

The difference between PriorPC with $\alpha = 1$ with PC-prior lies strictly in the tier-structure of the PriorPC. PriorPC outperforms PC-prior on all three datasets.

This section suggests that PriorPC wins on two aspects: the removal of the worst edges as a first step, and the use of prior knowledge. The first aspect is a nice result: it allows for faster processing and for larger networks, and clearly not at the expense of the accuracy. In fact removing these edges even helps the algorithm perform better tests. The second aspect shows that our inclusion of prior knowledge to rearrange the CI tests helps, even when given a low weight.

	DREAM4			*E. coli*			*B.subtilis*		
	TP	FP	F1	TP	FP	F1	TP	FP	F1
PC	86	678	0.18	314	5592	0.07	592	6725	0.12
PC-lite	80	206	0.35	127	369	0.09	361	1154	0.18
OPC	86	676	0.18	284	4534	0.08	561	5602	0.13
OPC-lite	79	208	0.34	126	366	0.09	342	1056	0.17
PC-prior	104	183	0.45	1355	435	0.70	1722	853	0.68
PriorPC $\alpha{=}0$	84	222	0.35	146	469	0.11	397	1199	0.19
PriorPC $\alpha{=}0.25$	100	188	0.43	253	452	0.18	545	1139	0.26
PriorPC $\alpha{=}0.5$	102	187	0.44	960	404	0.56	1025	988	0.46
PriorPC $\alpha{=}0.75$	106	171	0.47	1201	411	0.65	1522	756	0.64
PriorPC $\alpha{=}1$	106	173	0.47	1368	419	0.71	1748	813	0.70

Table 1: **From PC to PriorPC**. Effect of all the various steps between PC and PriorPC. None of the methods were subjected to bootstrapping. For PriorPC, all edges have a true prior. Two steps make a critical difference: discarding straight away the worst edges and using prior knowledge to rank the edges.

2 Threshold for conditional independenc test

Both PC and PriorPC require a threshold t for the CI tests as described in section 2.1 of the main article. Since we apply bootstrapping and use the frequency of occurrence to rank the edges, we choose t relatively small to have a graph contains a large number of edges. In this way, we keep more edges to compete. However, the threshold should not be too small which leads to computational singularity and require a lot of time or simply not be feasible.

In order to see the effect of the threshold t, we apply PC, PC-lite and PriorPC (with different values of α for incorporation of prior knowledge) for a variety of thresholds. Figure 1 shows the ROC curve and PR curve for Dream4 data, when we change the threshold t, $t \in \{0.001, 0.01, 0.1, 0.5, 1, 2\}$. The result shows that PC-lite and PriorPC with any value of α outperform the PC and this improvement is not the effect of bootstrapping. We did not tune the parameter t for each data set and set the parameter to 0.1 for all datasets.

3 Robustness to false prior

Biological prior knowledge can come from different sources including ChIP-seq data, protein-protein interaction data and literature, which can all contain false information. Methods for integrating prior knowledge should therefore be robust to errors.

In order to assess the robustness of the algorithms to erroneous prior information, we followed the experimental set up given in (?). 50% of the true edges were randomly selected and assigned a random

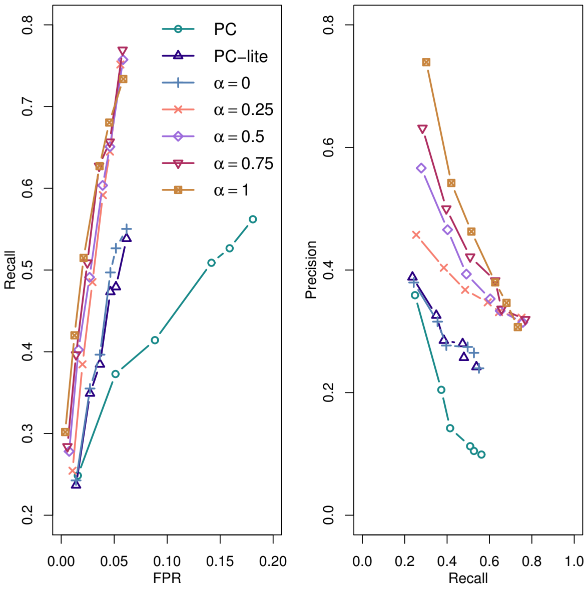

Figure 1: **Comparison of PC, PC-lite and PriorPC for DREAM4 data.** The left subplot shows the ROC curve, while the right subplot shows the PR curve for different tuning parameters. PriorPC (with any value of α) and PC-lite outperform the PC for all parameters.

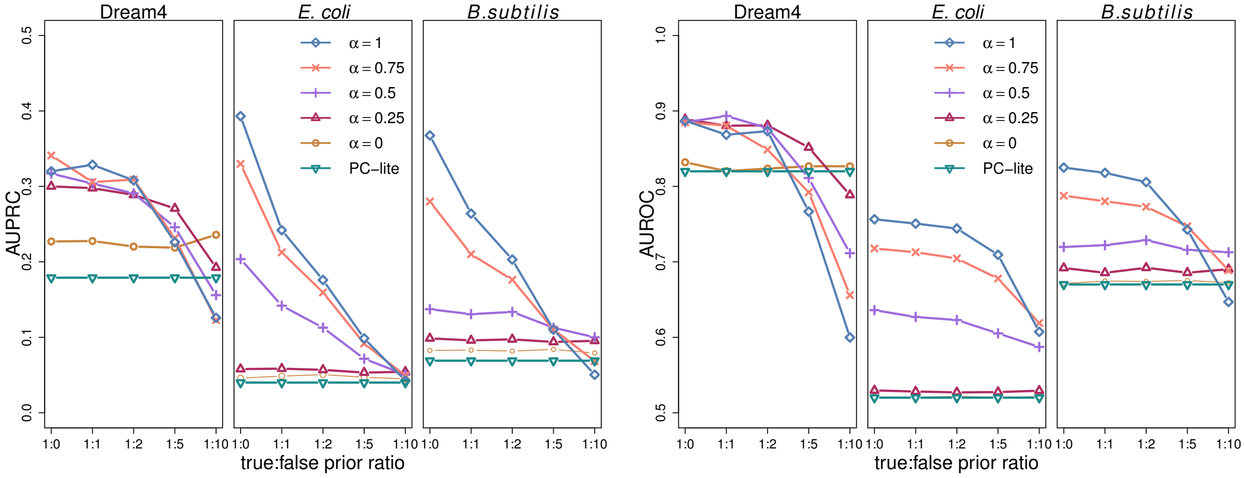

Figure 2: **Performance of PriorPC against the ratio of true priors to false priors.** The left subplot shows results for Dream4, the center subplot for E.coli and the right subplot for B.subtilis. A true prior is assigned to a random 50% of the edges present in the gold standard network. Different amounts of erroneous priors are then produced by flipping the true prior assigned to the remaining edges. The experiment is repeated for various values of α displayed in different colours. The performance of PC is plotted in green and with triangles for comparison. PriorPC performs better than PC-lite up to a ratio of true priors to false priors of 1:5.

prior higher than 0.5. Then m edges from the remaining edges in the prior matrix were selected and their corresponding true prior values were flipped so that $b_{ij} = 1 - b_{ij}$. Figure 2 shows the AUROC results for different ratio of true priors to false priors.

The results indicate that PriorPC is robust to reasonable amounts of error. Clearly, the higher the percentage of false priors, the worse the performance. Naturally, the results are less sensitive to errors for smaller values of α, which should be taken into account when choosing this parameter. Indeed, when α is small, PriorPC is still better than PC.
